# Juvenile vs. adult skeletal muscle transplants in the treatment of volumetric muscle loss injury

**DOI:** 10.1186/s13287-025-04844-y

**Published:** 2025-12-03

**Authors:** John J. Payne, Samuel R. Frandsen, Zachary H. Rasmussen, Matthew J. Mangus, Anna C. Taylor, Mason K. Kephart, Sandy S. Huang, Thomas K. Schiefer, Kyndal M. Jones, Erastus W. Evans, Jacob R. Sorensen

**Affiliations:** Department of Exercise Sciences, Brigham Young University, Provo, UT USA

**Keywords:** Volumetric muscle loss, Skeletal muscle transplantation, Satellite cells, Juvenile muscle, Donor age, Muscle regeneration, Tissue engineering, Stem cell therapy, Regenerative medicine

## Abstract

**Background:**

Volumetric muscle loss (VML) causes irreversible structural and functional deficits by removing myofibers, nerves, vasculature, extracellular matrix, and satellite cells, the resident muscle stem cells essential for regeneration. Skeletal muscle transplantation can restore tissue volume and reintroduce regenerative cells, yet functional outcomes remain incomplete. Age of the donor muscle has not been evaluated, despite evidence that juvenile muscle contains higher satellite cell density and greater myogenic plasticity than adult muscle. We hypothesized that these features would yield superior regenerative outcomes when juvenile muscle is used as a transplant source.

**Methods:**

Tibialis anterior (TA) muscles from juvenile (21 d), adolescent (34 d), and adult (~ 120 d) male Lewis rats were compared for myofiber morphology, satellite cell density, and in-vitro myogenic behavior. GFP⁺ juvenile or adult muscle was then transplanted into standardized VML defects (~ 15–20% TA volume) in adult rats. Seven weeks post-surgery, in-vivo isometric strength, donor fiber integration, satellite cell distribution, and centralized nuclei were assessed.

**Results:**

Juvenile muscle exhibited ~ 15× greater satellite cell density than adult (122.8 ± 28.4 vs. 8.4 ± 3.3 cells/mm², *p* < 0.0001) with enhanced in-vitro differentiation (fusion index + 73% vs. adult, *p* = 0.0067). In-vivo, both juvenile and adult transplants restored myofiber number to control levels (juvenile: 11,369 ± 1,511; adult: 9,115 ± 1,274; controls: 10,316 ± 685) and improved strength versus untreated VML (juvenile: +50%, *p* = 0.0016; adult: +36%, *p* = 0.0299). No significant functional differences were observed between donor ages. Donor fibers integrated but remained small, with localized satellite cell enrichment and increased centralized nuclei in transplant regions, consistent with ongoing regeneration.

**Conclusions:**

Juvenile skeletal muscle displays cellular and structural attributes favorable for regeneration and superior in-vitro myogenic behavior compared to adult muscle. However, these advantages did not translate into greater short-term in-vivo recovery following VML transplantation. Enhancing donor fiber hypertrophy, neuromuscular integration, and satellite cell expansion beyond the transplant region, potentially through rehabilitation or pharmaceutical interventions, may be necessary to realize the full therapeutic potential of juvenile donor muscle for regenerative medicine applications.

## Introduction

Volumetric muscle loss (VML) is a debilitating injury caused by trauma, tumor excision, or surgery that removes substantial amounts of skeletal muscle along with its cellular, vascular, neural, and extracellular matrix networks [1, 2]. This destructive injury eliminates structural support and depletes satellite cells, the resident muscle stem cells essential for regeneration [3, 4]. Because the satellite cell niche (basal lamina) is disrupted, the cells are unable to migrate into areas of damage and facilitate regeneration. Conversely, fibrotic scar tissue fills the void, overwhelming the intrinsic repair mechanisms, and results in chronic deficits in muscle strength and function [5, 6].

Clinically, there is no standard of care to treat VML, thus management is often focused on wound closure and limb salvage rather than restoration of muscle mass and function [7]. Experimental regenerative approaches, including skeletal muscle transplantation and autologous minced muscle grafts, aim to fill the defect with donor tissue that provides both structural scaffolding and a reservoir of regenerative cells [7, 8]. Upon transplantation, donor satellite cells can activate, proliferate, differentiate, and fuse with host fibers to support local regeneration [4]. However, even with successful donor integration, functional recovery is typically incomplete [5, 9], highlighting the potential need to optimize donor tissue characteristics.

One underexplored factor influencing transplant efficacy is the developmental age of donor muscle, as skeletal muscle undergoes pronounced structural and cellular changes across the lifespan [10–12]. Juvenile muscle exhibits small-diameter myofibers, high nuclear and satellite cell densities, and a microenvironment supportive of fiber growth and remodeling [3, 13, 14]. In contrast, adult muscle contains fewer satellite cells and larger, less plastic fibers [3, 10]. Such age-related differences may have critical implications for regenerative potential following transplantation.

Evidence from other regenerative contexts supports this concept: juvenile tissue or juvenile-like progenitors often outperform adult counterparts in repair, likely due to a more abundant and responsive stem cell pool combined with a growth-permissive environment [3, 10, 15]. Our group and others have shown that regenerative outcomes are constrained by the injury environment, including persistent denervation [16, 17], extracellular matrix remodeling [2], and limited stem cell migration into the defect [5]. These constraints suggest that the intrinsic advantages of juvenile donor tissue may still require complementary interventions, such as rehabilitation, to achieve full functional recovery [18–20].

Here, we tested the hypothesis that juvenile skeletal muscle, by virtue of its cellular composition and myogenic capacity, would outperform adult muscle in restoring structure and function following VML injury. We first characterized the morphology, satellite cell content, and in-vitro myogenic behavior of tibialis anterior (TA) muscle from juvenile, adolescent, and adult rats. We then compared the in-vivo regenerative outcomes of juvenile versus adult TA muscle transplants in a standardized rat VML model.

## Methods

This study was conducted in two parts. Study 1 evaluated the structural and cellular properties of juvenile, adolescent, and adult skeletal muscle to determine age-dependent differences relevant to regeneration. Study 2 examined the in-vivo regenerative performance of juvenile and adult muscle transplants in a rat model of VML, using functional and histological outcome measures seven weeks post-VML surgery. The work herein has been reported in line with the ARRIVE guidelines 2.0.

### Ethical approval

All experiments were approved by the Institutional Animal Care and Use Committee (IACUC) at Brigham Young University (Protocol #23–0401) in accordance with National Institutes of Health guidelines for the care and use of laboratory animals. All animals were housed on a 12–12 h light-dark cycle, with food and water ad libitum.

### Study 1: developmental analysis of donor muscle

#### Animals and tissue collection

To evaluate regenerative properties of skeletal muscle by age, male Lewis rats were grouped as juvenile (21 ± 0 days), adolescent (34 ± 0 days), or adult (130 ± 4.6 days) (*n* = 4 per group, 12 total). The left tibialis anterior (TA) muscle was harvested for histological analysis, and the right TA was used for myoblast isolation. Sample size was based on prior reports that show large age-related differences in rodent skeletal muscle. All animals were euthanized following tissue collection while under heavy isoflurane anesthesia (5%) until loss of reflexes, then bilateral thoracotomy was used as a secondary method to ensure death.

#### Immunohistochemistry and imaging

To assess age-related satellite cell content, myofiber number, and muscle morphology, TA muscles were collected, weighed, then embedded in tragacanth gum, snap frozen in isopentane, and cryosectioned (10 μm). Sections were fixed in 4% paraformaldehyde (Thermo Scientific), blocked with 10% normal goat serum (NGS; Thermo Fisher), and incubated overnight with Pax7 (mouse IgG1, 3 µg/mL; DSHB, AB_528428) and laminin (mouse IgG2a, 2 µg/mL; DSHB, AB_2618140). Secondary antibodies included Cy3 goat anti-mouse IgG1 (1:200; Jackson, 115–165−003) and Alexa Fluor 647 goat anti-mouse IgG2a (1:200; Jackson, 115–605−206). DAPI (1 µg/mL; Thermo Fisher, D1306) was used for nuclear counterstaining.

Whole tissue cross-sections were used to count myofibers and to measure whole TA and myofiber cross-sectional area. Images were acquired on a tile-scanning Echo Revolution fluorescence microscope using a 20× PlanX Apo objective. The laminin images were converted to grayscale and a threshold set (10–30 range) using Fiji (ImageJ) software. Myofibers were segmented using “Analyze Particles” with a size range of 50–8000 μm² and circularity 0.30–1.00. Manual correction was performed for incomplete borders. Mean fiber CSA and total fiber number were calculated per sample.

To assess satellite cell and nuclei content, five 1 mm² regions of interest were randomly imaged throughout each sample. Nuclei were counted based on images from the DAPI channel using the Otsu threshold parameters and the “watershed” feature to separate nuclei clusters. The “Analyze Particles” feature was set to a size threshold of 20–50 μm. Satellite cells were identified and counted manually based on Pax7⁺/DAPI⁺ co-localization within or near the laminin border.

#### Myoblast isolation and in vitro assays

To characterize age-dependent differences in satellite cell behavior and regenerative potential, primary myoblasts (activated satellite cells) were isolated from the right TA muscles of juvenile, adolescent, and adult rats. These cells were used in a series of in vitro assays to assess proliferative capacity, differentiation potential, and myogenic identity.

Right TA muscles were digested using the Miltenyi Skeletal Muscle Dissociation Kit and gentleMACS system. Cell suspensions were filtered (70 μm) and centrifuged, then incubated with PE-conjugated anti-CD106 (Miltenyi, 130-103-684) and sorted with Anti-PE MicroBeads (130-048-801) using LS columns. CD106⁺ myoblasts were seeded on Matrigel-coated plates and allowed to expand for 5–7 days in DMEM (Gibco) with 10% FBS and 1% penicillin-streptomycin changed every 48 h.

Myogenic identity was confirmed in passage 1 cells via MyoD1 staining (mouse IgG2b, 2 µg/mL; DSHB, AB_2146602) with Cy3 anti-IgG2b secondary (1:200; Jackson, 115-165-207). DAPI was used for nuclei staining. The percentage of MyoD⁺ nuclei was assessed in three random fields per group.

Myoblasts were seeded at a density of 25,000 cells/well on a Matrigel coated, 12-well plate and allowed to incubate overnight. Proliferation was assessed by EdU incorporation (Click-iT™ kit, Thermo Fisher, C10340) following manufacturer instructions at 4, 8, and 16 h. Briefly, cells were fixed (4% PFA), permeabilized (0.5% TritonX-100 in PBS), and labeled with Alexa Fluor 647-conjugated azide and Hoechst. Hoechst-stained nuclei were counted, and the proliferation index was calculated as EdU⁺ cells/total nuclei. Data were averaged from four fields per well, in triplicate wells per group.

For differentiation, cells were seeded at 100,000 cells per well on a Matrigel coated, 12-well plate and allowed to incubate overnight in growth media. Differentiation was induced by switching to DMEM + 2% horse serum for 72 h. Cells were fixed and stained for Myogenin (mouse IgG1, 2 µg/mL; DSHB, AB_2146601) and MyHC (mouse IgG2b, 2 µg/mL; DSHB, AB_2147781). Secondary antibodies included Alexa Fluor 647 anti-IgG1 and Cy3 anti-IgG2b (1:200; Jackson). DAPI was used for nuclear staining.

Three outcome measures were used to assess differentiation: fusion index, MyHC^+^ area, and nucleation index. The fusion index was defined as the percentage of total nuclei located within MyHC^+^ multinucleated myotubes, indicating the efficiency of myoblast fusion during early differentiation. Myotubes were defined as MyHC^+^ structures containing two or more DAPI-stained nuclei. The MyHC^+^ area was calculated as the total surface area occupied by MyHC^+^ staining within each field of view, measured in ImageJ using a threshold binary masks. The nucleation index was calculated as the average number of nuclei contained within each individual MyHC^+^ myotube. This index served as an indicator of myotube maturation. All quantifications were performed on at least four randomly selected, non-overlapping fields per well, and averaged across triplicate wells per condition.

### Study 2: VML injury and muscle transplantation

#### VML surgery and experimental design

To evaluate the regenerative performance of juvenile versus adult muscle transplants in a preclinical model of VML, a standardized full thickness VML injury and repair protocol was conducted in adult male rats. This approach was designed to mimic clinically relevant muscle trauma and assess functional and histological outcomes following transplantation of developmentally distinct donor tissue.

Twenty-four adult male Lewis rats (3–4 months old) were randomly assigned to one of three treatment groups: VML No Treatment, VML + Adult Transplant, or VML + Juvenile Transplant (*n* = 8 per group). Sample size was based on prior muscle transplant studies in a rat VML model to detect functional improvements of 20–40% [17]. All animals underwent unilateral VML surgery in the left TA muscle with the right leg serving as an uninjured, intra-animal control. To provide analgesia, a carprofen tablet (Bio-Serv, 5 g) was placed in the animal’s cage 24 h prior to surgery and a single preoperative dose of sustained-release buprenorphine (1.2 mg/kg, SC; Wedgewood) was injected subcutaneously into the back of the neck at least one hour before surgery.

Animals were anesthetized with 2–3% isoflurane in oxygen and placed in a supine position on a heated surgical platform. The lower left hindlimb was shaved and disinfected with alternating scrubs of chlorhexidine and 70% ethanol. A longitudinal skin incision (~ 1.5 cm) was made along the anterior surface of the lower leg to expose the underlying musculature. The skin and fascia were opened to expose the TA muscle, which was gently isolated from surrounding tissue using blunt dissection. To protect adjacent musculature, a sterile surgical spatula was inserted beneath the TA and full-thickness defect, approximately 6 mm in diameter (~ 15–20% of muscle volume), was created in the mid-belly region using a sterile biopsy punch (MedBlades, USA).

Donor tissue was harvested immediately prior to transplantation from ubiquitously expressing GFP⁺ juvenile (21-day; *n* = 4) or adult (~ 120-day; *n* = 2) male Lewis rats (Rat Resource and Research Center; Strain: LEW-Tg(CAG-EGFP)YsRrrc; RRRC#: 00206). TA muscles were dissected, placed in a sterile tissue culture dish on ice, and finely minced into ~ 1 mm³ fragments using sterile scissors. The total weight of minced tissue was adjusted to approximate the volume of the VML defect and then carefully implanted into the defect site.

For animals receiving transplants, the fascia was sutured, followed by subcutaneous closure of the skin using interrupted sutures. VML No Treatment animals underwent the same surgical procedure without tissue implantation.

#### In vivo muscle strength testing

To evaluate muscle function following VML injury and transplantation, in-vivo isometric force testing was performed seven weeks post-surgery for all of the VML injured limbs (*n* = 24) and most (*n* = 19) of the uninjured control limbs. Under 2–3% isoflurane anesthesia, rats were placed supine on a temperature-controlled platform. The hindlimb was immobilized using a knee clamp, and the foot was secured to a force transducer footplate (Aurora Scientific 3-in-1 Muscle Test System).

Subcutaneous needle electrodes were inserted near the peroneal nerve to stimulate the anterior compartment (TA/EDL) of the hindlimb. Stimulation parameters included 0.1 ms pulse width, 400 ms train duration, and increasing frequencies ranging from 10 to 200 Hz. Optimal voltage was determined for each animal to ensure maximal contractile response. Peak isometric tetanic force was recorded as the highest value generated during the frequency ramp. To account for inter-animal variability in body size, absolute force values were normalized to body weight (mN·m/kg).

#### Tissue collection and histology

Seven weeks post VML surgery, TA, EDL, soleus, and gastrocnemius muscles were dissected and weighed. Animals were then euthanized with an overdose of isoflurane (5%) and bilateral thoracotomy as a secondary measure to ensure death. TA sections were frozen in isopentane cooled in liquid nitrogen and stored at − 80 °C. The TA muscles were sectioned (10 μm) and stained for laminin, Pax7, and DAPI. High-resolution images were taken randomly from controls and from three regions in VML injured samples: defect/transplant region (typically devoid of host myofibers or identified by GFP expression), border (adjacent to the injury; smaller, disorganized host fibers), and distal (intact muscle away from the injury site). Satellite cells were counted based on Pax7⁺/DAPI⁺ co-localization. Myofiber CSA, total fiber count, and centrally nucleated fibers were quantified from regions of interest using ImageJ analysis software and the previously described segmentation thresholds.

#### Donor fiber analysis

To assess transplant integration and morphology, TA muscle sections were analyzed for GFP fluorescence and labeled with wheat germ agglutinin (WGA) to identify myofiber boundaries. Sections were cut at 10 μm thickness and immediately fixed in 2% paraformaldehyde for 10 min at room temperature to preserve endogenous GFP signal. Following fixation, slides were rinsed once with PBS and incubated for 15 min with Alexa Fluor 647-conjugated wheat germ agglutinin (WGA; 1:500 dilution in PBS; Thermo Fisher Scientific, Cat# W32466). Sections were washed once in PBS and mounted in Fluoroshield (Sigma) and imaged using a 20X PlanX Apo objective on the Echo Revolution fluorescence microscope. GFP fluorescence was visualized directly without antibody amplification. Exposure settings were optimized to avoid saturation and kept constant between samples within each group.

To quantify GFP⁺ donor fibers, high-resolution images were collected from well-defined GFP regions within the transplant zone. Fields with high signal-to-noise ratio and clear membrane borders were prioritized for analysis. Myofiber CSA was measured using ImageJ. GFP-positive fibers were defined as those showing continuous cytoplasmic GFP signal enclosed by WGA-labeled borders. A minimum of 200 GFP-positive fibers were analyzed per animal. The GFP area was also calculated as a percentage of total CSA using full-section scans in ImageJ, thresholded for GFP fluorescence.

#### Statistical analysis

All statistical analyses were performed using GraphPad Prism version 10.5.0. One-way ANOVA with Tukey’s post hoc test was used for group comparisons. Data are presented as mean ± SD. A p-value < 0.05 was considered statistically significant.

## Results

### Study 1: comparison between juvenile, adolescent, and adult male Lewis rats

#### Rat characteristics

Juvenile (21-day), adolescent (34-day), and adult (120-day) male Lewis rats were used to represent distinct developmental stages. As expected, body and TA muscle weights increased significantly with age (Table 1), consistent with known patterns of postnatal growth.

**Table 1:** Rat characteristics

Group	Age (days)	Body weight (grams)	TA wet weight (grams)
Juvenile	21 ± 0^a, b^	53.85 ± 0.37^a, b^	0.16 ± 0.0036^a, b^
Adolescent	34 ± 0^a^	144.5 ± 6.25^a^	0.25 ± 0.0183^a^
Adult	120 ± 5.7	396.5 ± 16.6	0.775 ± 0.031

a = significant difference from adult. b = significant difference from adolescent (*p* ≤ 0.01)

#### Muscle size and myofiber architecture vary by developmental stage

**Fig. 1:**
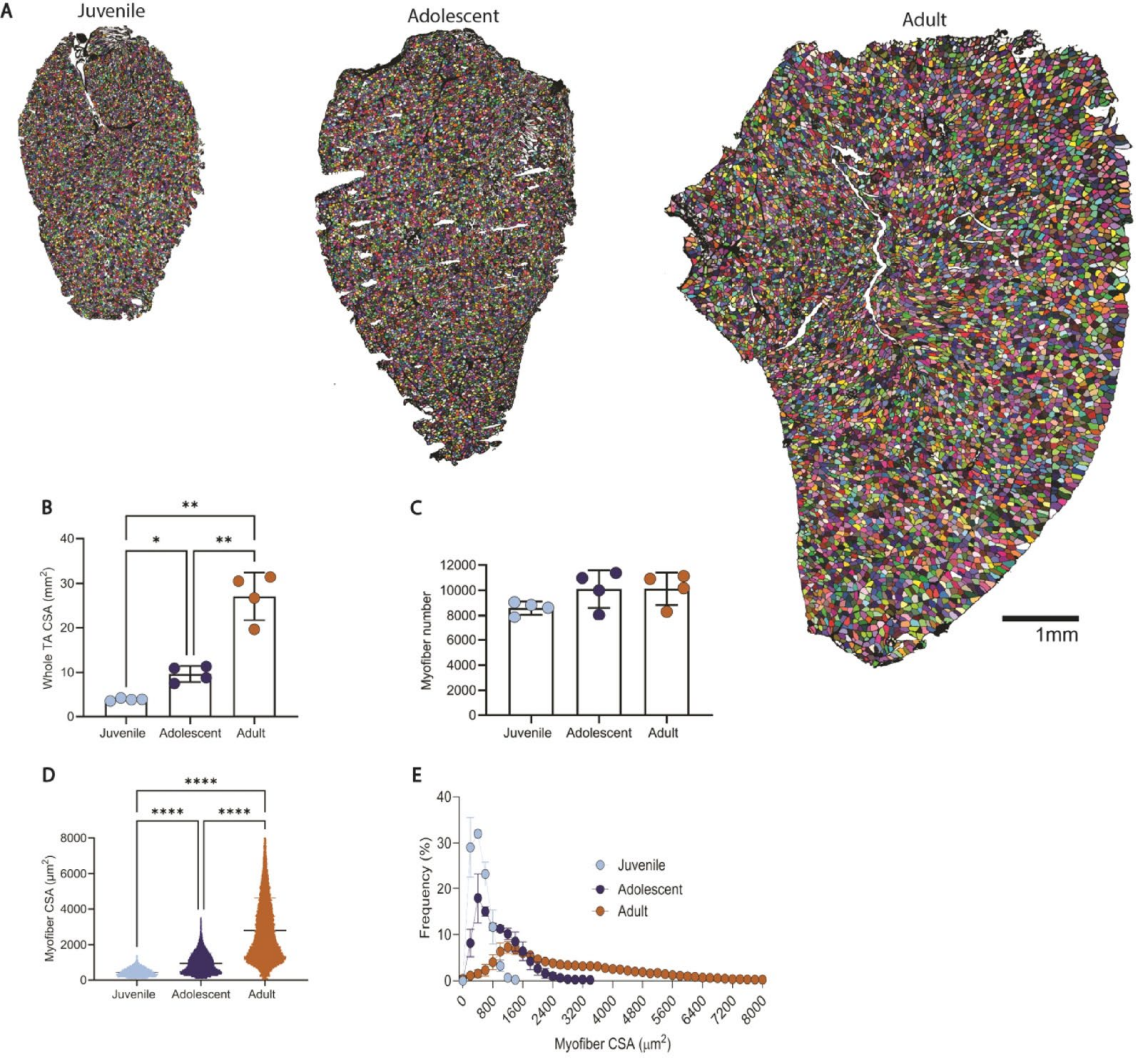
Tibialis anterior (TA) muscle structure and myofibers vary by age. (**A**) Representative transverse cross-sections of the tibialis anterior (TA) muscle from juvenile (21-day), adolescent (34-day), and adult (120-day) male Lewis rats. Individual myofibers are pseudo-colored based on segmentation from laminin staining. (**B)** Whole-muscle CSA increased stepwise with age, more than doubling from juvenile to adolescent (+144%, *p* = 0.0012) and nearly tripling again from adolescent to adult (+181%, *p* = 0.0003). Adult CSA was ~7-fold greater than juvenile (*p* < 0.0001). (**C**) Total myofiber number per muscle did not significantly differ between groups (*p*=0.1621), indicating growth was primarily driven by fiber hypertrophy rather than hyperplasia. (**D**) Average myofiber CSA increased ~2-fold from juvenile to adolescent (*p* = 0.0267) and nearly 3-fold again from adolescent to adult (*p* < 0.0001). Adult fibers were more than 6-fold larger than juvenile (*p* < 0.0001). (**E**) Frequency distribution of myofiber CSA further illustrates that juvenile muscle exhibited a narrow, left-shifted distribution, while adolescent and adult muscles showed progressively broader and right-shifted profiles. Data are presented as mean ± SD; *n* = 4 per group. Statistical significance: ****p < 0.0001, **p < 0.01, *p < 0.05. Scale bar = 1 mm

Whole-muscle CSA increased significantly with age (Fig. 1A, B). Juvenile TAs averaged 3.95 ± 0.29 mm², adolescent TAs 9.64 ± 1.80 mm², and adult TAs 27.07 ± 5.36 mm². All pairwise comparisons were statistically significant (juvenile vs. adolescent *p* = 0.0012; juvenile vs. adult *p* < 0.0001; adolescent vs. adult *p* = 0.0003), confirming stepwise muscle growth. Despite these CSA increases, total myofiber number did not significantly differ between groups (juvenile: 8593 ± 525; adolescent: 10,101 ± 1484; adult: 10,126 ± 1282; *p* = 0.1621), indicating that muscle growth was driven primarily by fiber hypertrophy, not hyperplasia (Fig. 1C).

Analysis of individual myofiber CSA revealed age-related increases (Fig. 1D). Juvenile fibers averaged 460 ± 38 μm², significantly smaller than adolescent (963 ± 147 μm²; *p* = 0.0267) and adult fibers (2798 ± 354 μm²; *p* < 0.0001). Adolescent fibers were also significantly smaller than adult fibers (*p* < 0.0001). The CSA frequency distributions further emphasized this shift as juvenile muscle showed a narrow peak at small fiber sizes, whereas adolescent and adult profiles were broader and right-shifted, consistent with increased fiber size (Fig. 1E).

#### Nuclei, myofiber, and satellite cell density decline with advancing age

**Fig. 2:**
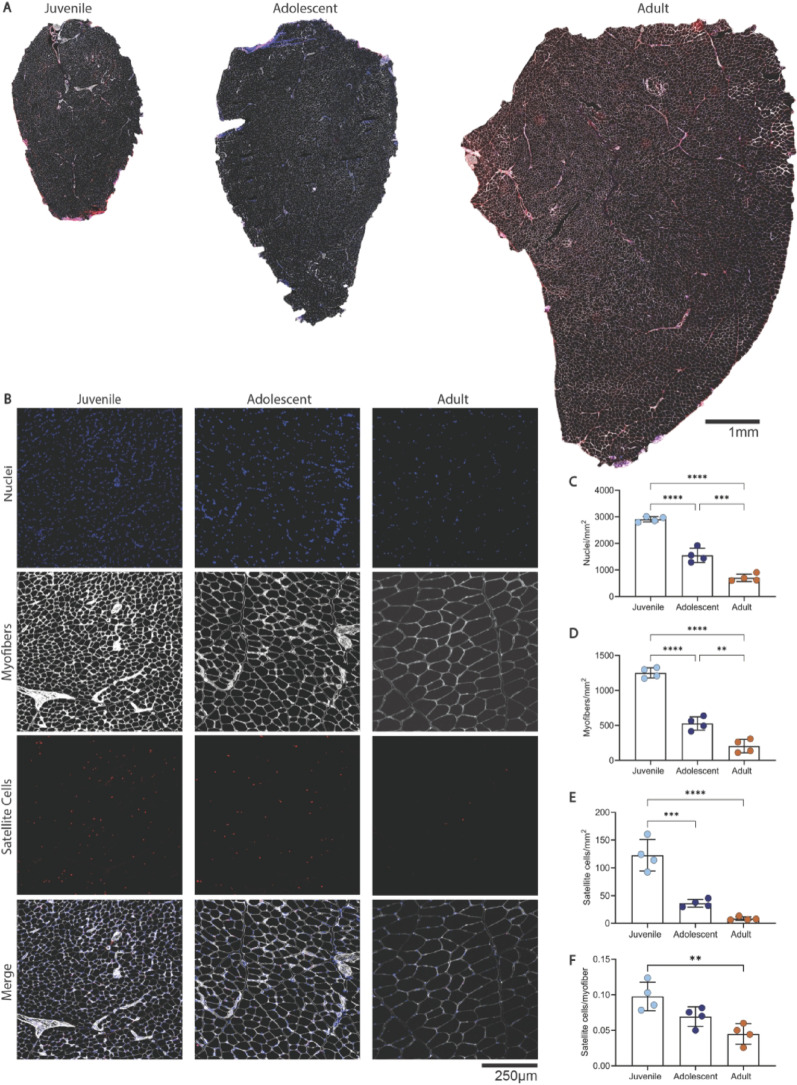
Nuclei, myofiber, and satellite cell density decline with age. (**A**) Representative whole-muscle cross-sections of the tibialis anterior (TA) from juvenile (21-day), adolescent (34-day), and adult (120-day) male rats. (**B**) High-magnification images show DAPI-stained nuclei (blue), laminin-labeled myofiber membranes (white), and Pax7⁺ satellite cells (red). (**C**) Quantification of nuclear density (nuclei/mm²) revealed a significant stepwise decline with age: adolescents had ~47% fewer nuclei than juveniles (*p* < 0.0001), and adults had ~55% fewer than adolescents (*p* < 0.0001), leaving adult muscle with less than one-quarter of juvenile density overall. (**D**) Myofiber density (fibers/mm²) also decreased significantly with age, dropping ~50% from juvenile to adolescent (*p* < 0.001) and another ~50% from adolescent to adult (*p* < 0.001), consistent with growth by fiber hypertrophy. (**E**) Satellite cell density (Pax7⁺ cells/mm²) was ~3.5-fold higher in juveniles compared to adolescents (*p* < 0.001) and ~15-fold higher than in adults (*p* < 0.0001). Although adolescent satellite cell density appeared greater than adult, this difference did not reach significance (*p* = 0.1049). (**F**) When normalized to myofiber number, juvenile tissue maintained a 2-fold increase in satellite cells compared to adults (*p* = 0.0035). Data are presented as mean ± SD, *n* = 4 animals per group. Statistical comparisons were made using one-way ANOVA followed by Tukey’s post hoc test. Statistical significance: ****p < 0.0001, ***p < 0.001, **p < 0.01. Scale bars = 1 mm (**A**), 250 μm (**B**)

To assess how developmental age affects muscle, we quantified nuclei, myofiber density, and Pax7⁺ satellite cells per mm² (Fig. 2A–B). Satellite cell and myofiber counts were normalized to tissue area to reflect volumetric density. This approach provides a physiologically relevant comparison, as it indicates that juvenile muscle contains a higher number of fibers and nuclei per unit volume, suggesting that, for a given VML defect size, juvenile tissue would contribute substantially more regenerative cells and fibers than adult tissue. Nuclear density was highest in juvenile muscle (2907.5 ± 99.4 nuclei/mm²), declined to 1550.4 ± 268.1 in adolescents, and dropped further in adults (704.3 ± 139.6; *p* < 0.0001 for all comparisons) (Fig. 2C). Myofiber density also declined significantly with age (*p* < 0.001), consistent with increasing fiber size (Fig. 2D).

Satellite cell density, measured by Pax7⁺ nuclei, showed a dramatic age-related decline (Fig. 2E, F). Juvenile muscles contained 122.8 ± 28.4 Pax7⁺ cells/mm², approximately 3.5 times higher than adolescents (36.1 ± 7.1; *p* < 0.001) and 15 times higher than adults (8.4 ± 3.3; *p* < 0.0001). Although adolescent satellite cell density appeared higher than adult, the difference did not reach statistical significance (*p* = 0.1049). To account for the age-related increase in myofiber size, satellite cells were also expressed relative to myofiber number (Pax7⁺ cells/myofiber). This normalization maintained the same pattern, with juveniles averaging 0.097 ± 0.009 Pax7⁺ cells/fiber, adolescents 0.069 ± 0.006, and adults 0.045 ± 0.008. Even after adjusting for fiber size, juvenile muscle retained roughly twice as many satellite cells per myofiber as adult tissue (*p* = 0.0035), confirming that the higher cellularity reflects genuine enrichment beyond differences in muscle architecture.

#### Myoblast proliferation and differentiation

To assess how donor age affects cellular behavior, myoblasts were isolated from juvenile, adolescent, and adult TA muscles and evaluated in-vitro.

#### Myogenic purity and proliferation

**Fig. 3:**
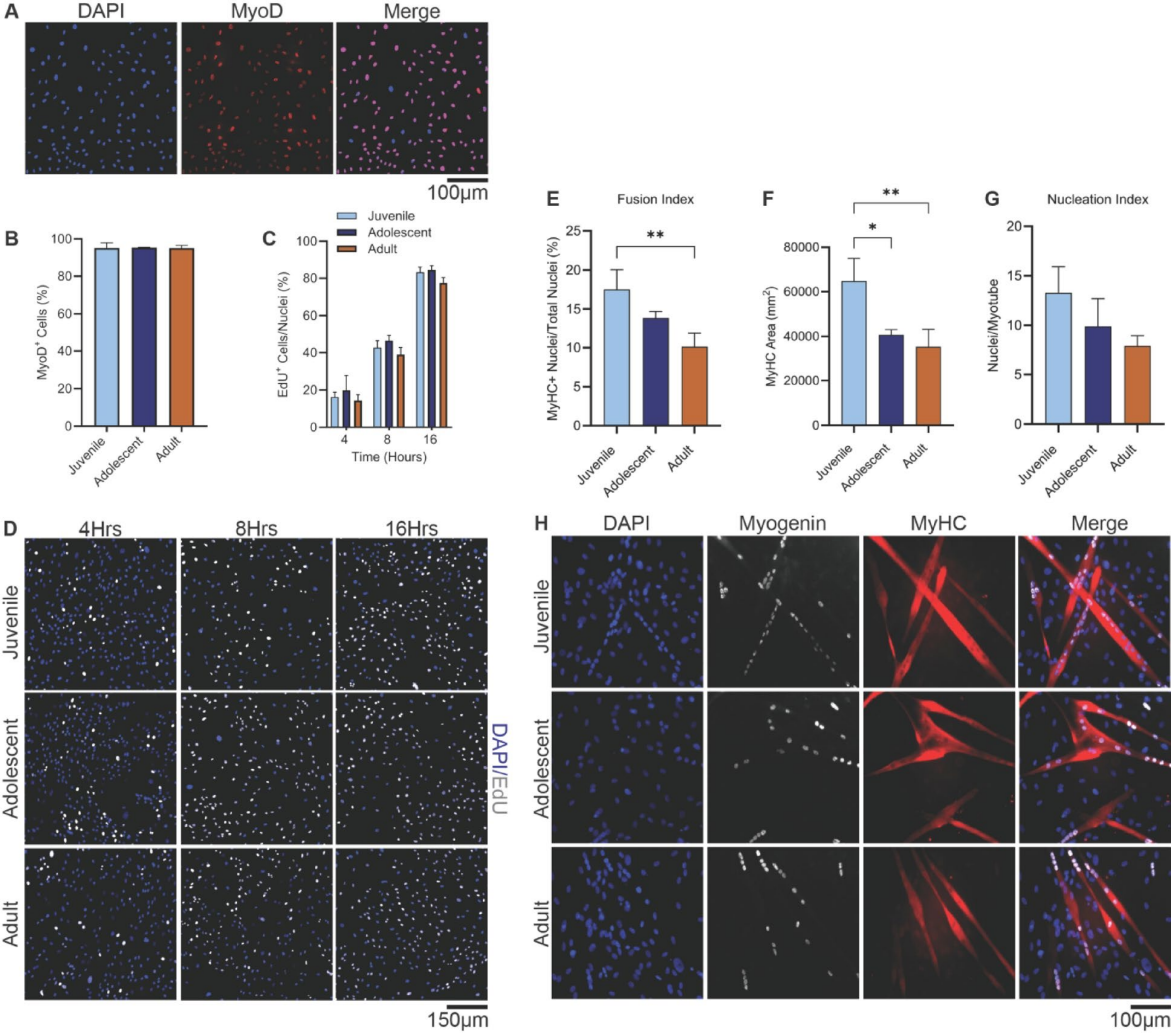
Myoblast purity, proliferation, and differentiation across donor age groups. (**A**) Representative immunofluorescence image from an adult-derived myoblast culture showing co-localization of nuclear MyoD (red) with DAPI-stained nuclei (blue), confirming myogenic identity. (**B**) Quantification of MyoD⁺ nuclei indicated >95% purity in cultures from juvenile, adolescent, and adult donors, confirming successful isolation of committed myogenic cells. (**C**) EdU incorporation increased from ~15% at 4 h to ~80% by 16 h in all groups, with no age-related differences (*p* = 0.9696), indicating a similar proliferative capacity. Representative images (**D**) of DAPI (blue) and EdU (white) staining illustrate comparable proliferative activity among juvenile, adolescent, and adult myoblasts at each time point. (**E**) After 72 h of differentiation, the fusion index (MyHC⁺ nuclei/total nuclei) was ~73% higher in juvenile vs. adult myoblasts (*p*=0.0067). Adolescent cultures were intermediate and not significantly different from either group (*p*>0.1). (**F**) Total myotube formation, measured by MyHC⁺ area per field, was ~60–80% larger in juvenile compared to adolescent (*p*=0.0191) and adult (*p*=0.0074) myoblasts, respectively. No difference was observed between adolescent and adult groups (*p* = 0.6658). (**G)** The nucleation index (nuclei/myotube) did not differ significantly across groups (*p*>0.05). (**H**) Representative images of differentiated myoblasts stained for DAPI (blue), Myogenin (white), and MyHC (red) highlight enhanced multinucleated myotube formation in juvenile cultures. Data are presented as mean ± SD. Values reflect the mean of 4–5 images per well, collected in triplicate wells (*n* = 3) for each group at each time point. Statistical significance: **p < 0.01, *p < 0.05. Scale bars = 100 μm (**A**, **H**), 150 μm (**D**)

Immunostaining confirmed high myogenic purity across all groups, with MyoD⁺ nuclei accounting for 95.3 ± 2.7% in juvenile, 95.4 ± 0.2% in adolescent, and 95.2 ± 1.4% in adult cultures (Fig. 3A, B). EdU incorporations over 4, 8, and 16 h showed a time-dependent increase in proliferation, rising from ~ 15% at 4 h to ~ 80% at 16 h across all groups. However, no significant differences in proliferation rate were observed between age groups at any time point (*p* = 0.9696), indicating that donor age did not affect baseline proliferative capacity (Fig. 3C, D).

#### Enhanced differentiation in juvenile myoblasts

In contrast, differentiation declined significantly with age. After 72 h in differentiation medium, juvenile myoblasts showed a higher fusion index (17.49 ± 2.53%) than adolescent (13.86 ± 0.83%) and adult (10.12 ± 1.79%) cultures. The difference between juvenile and adult groups was statistically significant (*p* = 0.0067), while differences between juvenile and adolescent (*p* = 0.1161) and adolescent and adult (*p* = 0.1066) were not (Fig. 3E).

Myosin Heavy Chain (MyHC) area per field, a measure of total myotube formation, was significantly larger in juvenile cultures (64,667 ± 10,161 μm²) than in adolescent (40,652 ± 2,331 μm²; *p* = 0.0191) and adult cultures (35,149 ± 7,968 μm²; *p* = 0.0074) (Fig. 3F). No significant difference was observed between adolescent and adult groups (*p* = 0.6658).

The nucleation index, defined as the average number of nuclei per myotube, did not differ significantly between groups. Juvenile (13.27 ± 2.62), adolescent (9.89 ± 2.79), and adult (7.88 ± 1.07) cultures exhibited similar nuclear content per fiber, with no statistically significant differences (Fig. 3G). Overall, these findings indicate that juvenile myoblasts have an enhanced capacity for differentiation and myotube formation, despite equivalent proliferation.

### Study 2: comparison between VML treatment groups

#### Rat characteristics at time of VML surgery

To evaluate transplant performance, rats were randomized to VML No Treatment, VML + Adult Transplant, or VML + Juvenile Transplant groups (*n* = 8/group). All animals were of similar age and body weight at the time of surgery, with no significant differences between groups (Table 2). However, the VML tissue excised from the No Treatment group was significantly smaller than in the transplant groups, which may have contributed to milder deficits in untreated animals. Transplant weights did not differ significantly between juvenile and adult donor groups.

**Table 2:** Rat characteristics at time of surgery

Group	Age at surgery (Days)	Weight at surgery (g)	Weight of VML piece (mg)	VML transplant weight (mg)
VML no treatment	110.63 ± 3.62	385.13 ± 32.52	73.97 ± 12.89^a, b^	N/A
VML + Adult Transplant	113.00 ± 11.78	418.88 ± 42.29	85.79 ± 18.03	93.94 ± 20.79
VML + Juvenile Transplant	107.30 ± 14.95	404.20 ± 19.23	83.40 ± 14.17	97.60 ± 11.07

a = significant difference from adult. b = significant difference from adolescent (*p* ≤ 0.05)

#### Rat characteristics post VML surgery

At tissue collection (seven weeks post-surgery), body weight was lower in the No Treatment group (415.3 ± 14.6 g) compared to the Adult (440.9 ± 38.8 g) and Juvenile (443.6 ± 25.0 g) transplant groups (*p* < 0.05). Despite this, individual muscle wet weights, including the TA, EDL, soleus, and gastrocnemius, were not significantly different across groups (Table 3), suggesting that gross muscle mass was not markedly influenced by transplantation within the time window assessed.

**Table 3:** Muscle weights

		Wet Weight (g)							
Group	Body Weight at Collection (g)	Right TA	Left TA	Right EDL	Left EDL	Right Soleus	Left Soleus	Right Gastroc	Left Gastroc
VML No Treatment	415.29 ± 14.57^a, b^	0.74 ± 0.07	0.70 ± 0.12	0.20 ± 0.04	0.20 ± 0.02	0.17 ± 0.04	0.19 ± 0.04	1.90 ± 0.12	1.99 ± 0.21
VML + Adult Transplant	440.86 ± 38.84	0.77 ± 0.03	0.73 ± 0.09	0.17 ± 0.02	0.17 ± 0.02	0.21 ± 0.03	0.17 ± 0.01	1.99 ± 0.14	1.96 ± 0.08
VML + Juvenile Transplant	443.56 ± 24.97	0.75 ± 0.11	0.78 ± 0.15	0.17 ± 0.01	0.20 ± 0.03	0.18 ± 0.04	0.19 ± 0.03	1.90 ± 0.15	2.00 ± 0.15

Tibialis Anterior (TA), Extensor Digitorum Longus (EDL), Gastrocnemius (Gastroc). a = significant difference from adult transplant. b = significant difference from juvenile transplant (*p* ≤ 0.05)

#### In-vivo strength measurements

##### Functional recovery

To assess whether muscle transplantation improved force production, in-vivo isometric strength of the TA/EDL complex was measured in most of the uninjured right limbs (controls; *n* = 19) and all of the VML injured limbs (*n* = 24) seven weeks post-injury. Uninjured control limbs generated the highest absolute force (20.96 ± 3.04 mN·m, *n* = 19), significantly greater than all VML-injured groups (*p* < 0.0001). No Treatment animals exhibited the lowest force output (9.47 ± 0.67 mN·m, *n* = 8). Adult transplants restored force to 12.94 ± 1.52 mN·m (*n* = 8; *p* = 0.0299 vs. No Treatment), while juvenile transplants reached 14.25 ± 2.01 mN·m (*n* = 8; *p* = 0.0016 vs. No Treatment), though not significantly greater than adult (*p* = 0.6992).

Normalized to body weight, a similar pattern emerged. Control limbs produced 48.12 ± 6.44 mN·m/kg, while VML No Treatment animals fell to 23.19 ± 2.50. Juvenile and adult transplants improved force to 32.16 ± 4.42 and 29.12 ± 4.53 mN·m/kg, respectively. Only juvenile transplants produced a statistically significant improvement over No Treatment (*p* = 0.0141), while adult transplants did not (*p* = 0.1670). No significant difference was observed between transplant groups (*p* = 0.7025). These data suggest that both donor types support partial recovery of force, with juvenile transplants trending slightly higher.

#### Histological assessment of regeneration

**Fig. 4:**
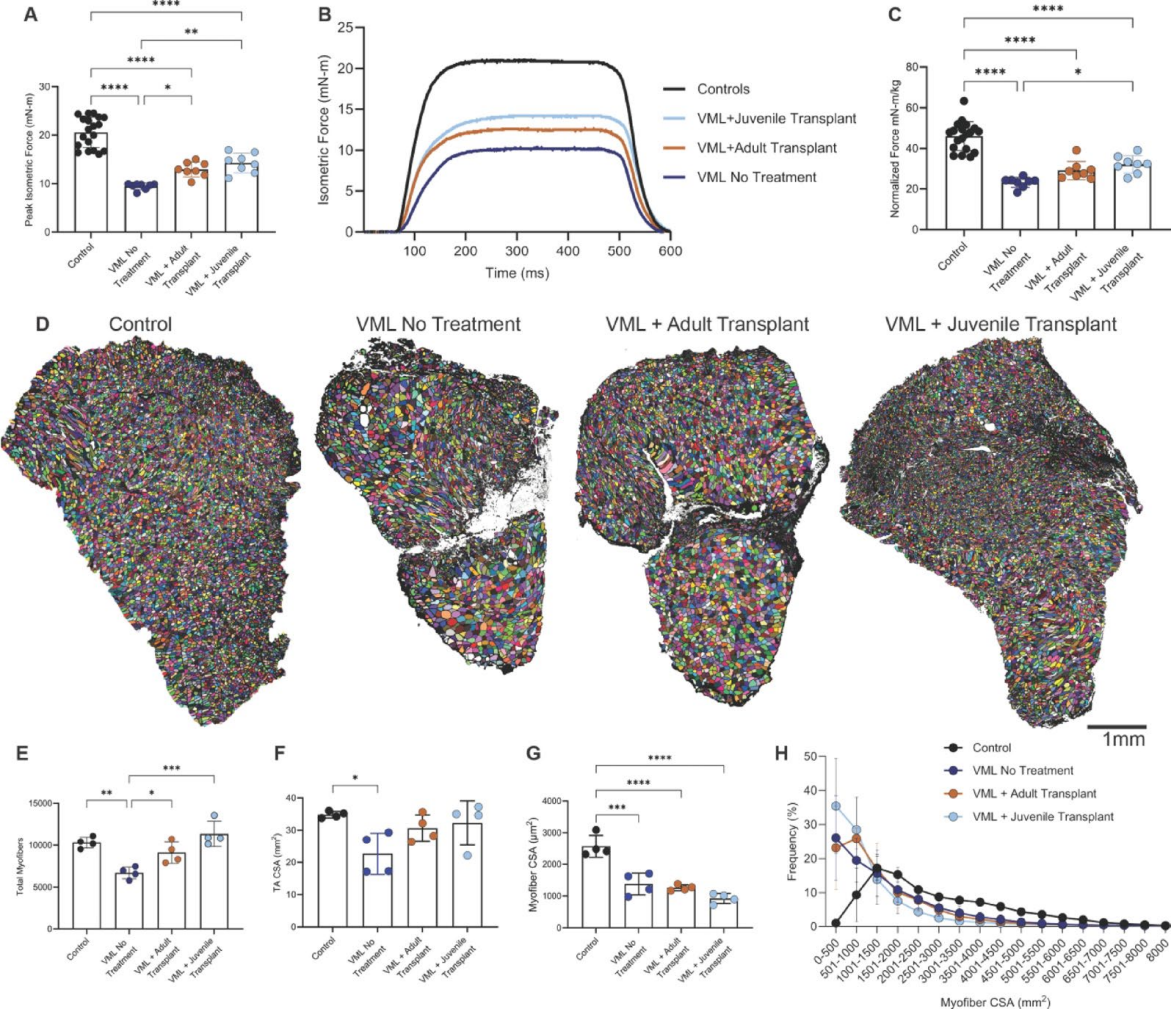
Muscle force and myofiber morphology following VML injury and muscle transplantation. In vivo strength testing of the anterior compartment (tibialis anterior and extensor digitorum longus) revealed significant force deficits following VML, with partial recovery after transplantation. Peak isometric force **(A)** was highest in controls (20.96 ± 3.04 mN·m, *n*=19) and reduced by ~55% in untreated VML limbs (9.47 ± 0.67, *n*=8). Adult transplants restored force to 12.94 ± 1.52 (*n*=8; *p* = 0.0299 vs. VML No Treatment), and juvenile transplants reached 14.25 ± 2.01 (*n*=8; *p* = 0.0016 vs. VML No Treatment), representing a 36% and 50% increase, respectively (Adult vs. Juvenile, *p* = 0.6992). Force traces **(B)** illustrate these differences. Body-weight–normalized force **(C)** showed a similar pattern. Histological analysis was performed on a subset of animals to explain differences in force (*n* = 4 per group). Representative TA cross-sections **(D)** show severe tissue loss in untreated VML, while both transplant groups showed improved fiber retention but smaller fibers. Each color represents a pseudo-colored myofiber based on laminin staining. Myofiber quantification **(E–H)** supported these observations. Fiber number **(E)** was significantly reduced in untreated VML muscle compared to controls (*p* < 0.01), while both transplant groups restored myofibers, with juvenile recipients ~25% higher than adults. Total TA CSA **(F)** was also reduced in the No Treatment muscle (*p* = 0.0265 vs. control) but preserved in both transplant groups (≥85% of control). Mean myofiber CSA **(G)** remained significantly smaller in all VML groups vs. controls (*p* < 0.001), reflecting persistent immature fibers. The myofiber CSA distributions **(H)** showed a leftward shift toward small fibers (<1,000 μm²) in all VML groups, most pronounced in juveniles, suggesting ongoing regeneration but with limited hypertrophy in the transplanted tissue. Data are mean ± SD. Statistical significance: ****p < 0.0001, ***p < 0.001, **p < 0.01, *p* < 0.05. Scale bar = 1 mm

Transverse TA sections were analyzed in a subset of animals from each group (*n* = 4) to evaluate fiber number and muscle size, representing fiber maturity and regeneration. Total myofiber number was significantly reduced in the No Treatment group (6,952 ± 743) compared to uninjured controls (10,316 ± 685; *p* < 0.01). Transplants restored fiber number, with adult recipients averaging 9115 ± 1274 fibers and juvenile recipients 11,369 ± 1,51, which were comparable to controls (Fig. 4E). TA muscle CSA was lowest in the No Treatment group (22.7 ± 5.5 mm²), significantly smaller than controls (34.3 ± 1.0 mm²; *p* = 0.0265). Transplantation preserved CSA (adult: 30.6 ± 3.6 mm²; juvenile: 32.7 ± 6.6 mm²), as neither group differed significantly from controls (Fig. 4F).

Despite improvements in fiber number and muscle size, the average myofiber CSA remained significantly reduced in all VML groups. Controls averaged 2569 ± 334 μm², compared to 1379 ± 290 (No Treatment), 1,258 ± 97 (adult transplant), and 923 ± 151 μm² (juvenile transplant) (Fig. 4G). CSA distribution curves showed that juvenile transplants had the highest proportion of small-diameter fibers (< 1000 μm²), suggesting ongoing regeneration or limited hypertrophy of transplanted fibers at this potentially early time point (Fig. 4H).

#### Integration of GFP⁺ donor fibers

**Fig. 5:**
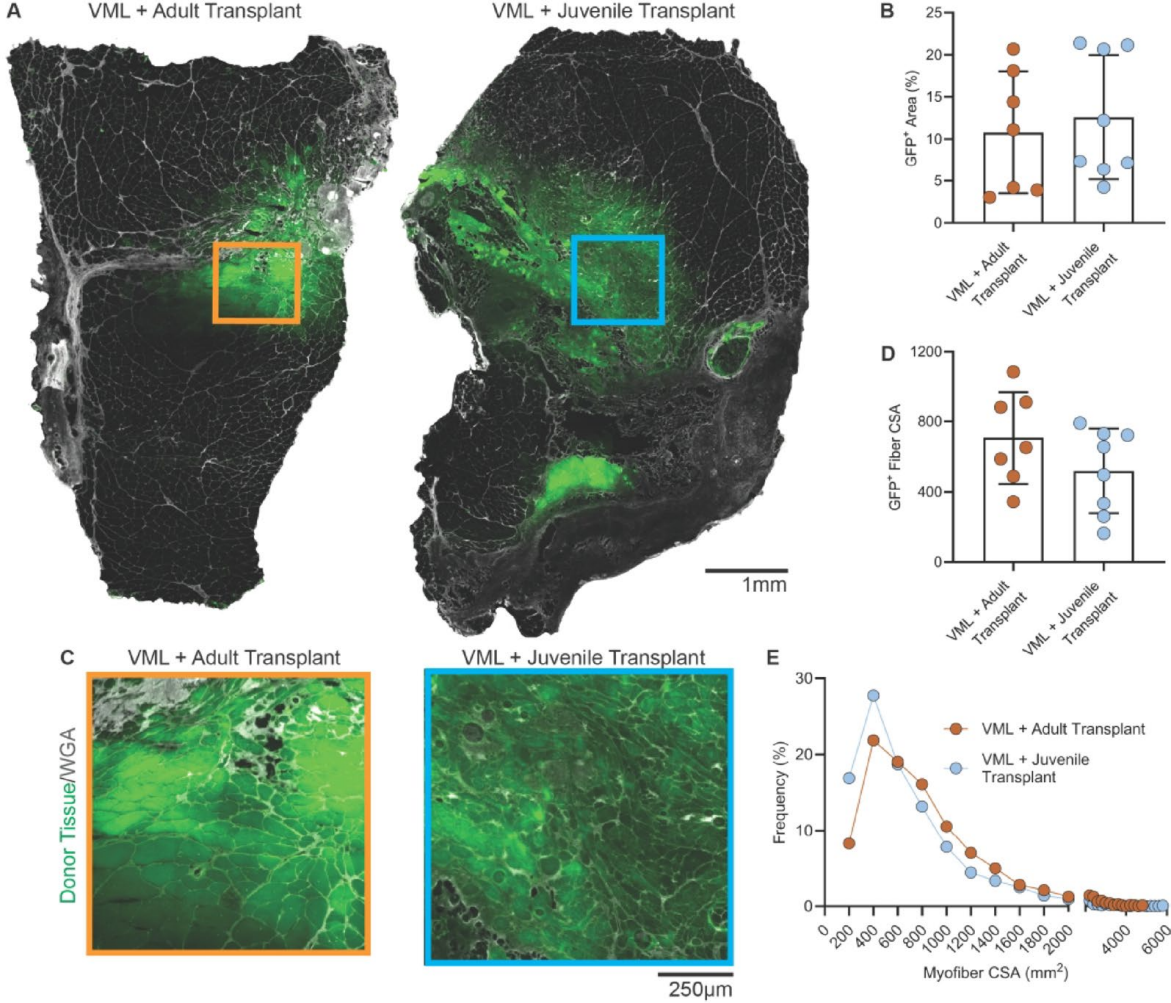
Donor fiber (GFP^+^) integration and morphology following juvenile and adult muscle transplantation. Seven weeks after volumetric muscle loss (VML) injury and transplantation with GFP⁺ donor muscle, cross-sectional imaging of the TA muscle revealed localized integration of donor tissue within the transplant region. Whole-muscle sections **(A)** show GFP⁺ signal (green) corresponding to donor cells, overlaid with wheat germ agglutinin (WGA; white) to visualize overall muscle structure. Both adult and juvenile transplant groups demonstrated similar spatial localization and extent of GFP⁺ signal. Quantification **(B)** showed GFP⁺ tissue occupied 10.78 ± 7.27% of total TA CSA in adult recipients (*n*=7) and 12.57 ± 7.39% in juvenile recipients (*n* = 8), with no significant difference between groups (*p*=0.6412). High magnification views **(C)** highlight individual GFP⁺ myofibers surrounded by WGA-stained extracellular matrix. Analysis of GFP⁺ myofiber CSA **(D)** indicated mean donor fibers were significantly smaller than control host fibers (*p* < 0.001), averaging <1,000 μm² in both transplant groups, with no significant difference between adult and juvenile means (*p*>0.05). Distribution analysis **(E)** revealed >70% of GFP⁺ fibers were <1,000 μm², consistent with regenerating or immature myofibers. A modest leftward shift was detected in the juvenile group, reflecting a higher proportion of very small donor fibers. Data are presented as mean ± SD. Scale bars = 1 mm (**A**), 250 μm (**C**)

To assess integration of transplanted tissue, GFP fluorescence was analyzed. All but one transplant recipient displayed a clear GFP⁺ region within the injury zone (Fig. 5A–C). GFP⁺ area occupied 10.78 ± 7.27% of the total muscle CSA in adult recipients (*n* = 7) and 12.57 ± 7.39% in juvenile recipients (*n* = 8), with no significant difference between groups (*p* = 0.6412; Fig. 5D).

Average CSA of GFP⁺ fibers was smaller in juvenile transplants (518.6 ± 230.7 μm²; *n* = 8) than in adult transplants (707.9 ± 246.6 μm²; *n* = 7), though this difference did not reach statistical significance (*p* = 0.1735). Distribution profiles confirmed that juvenile transplants contained a higher proportion of small-diameter GFP⁺ fibers, while adult transplants included a broader range of sizes (Fig. 5E). These patterns likely reflect developmental differences in donor fiber size and post-transplant remodeling.

#### Satellite cell distribution

**Fig. 6:**
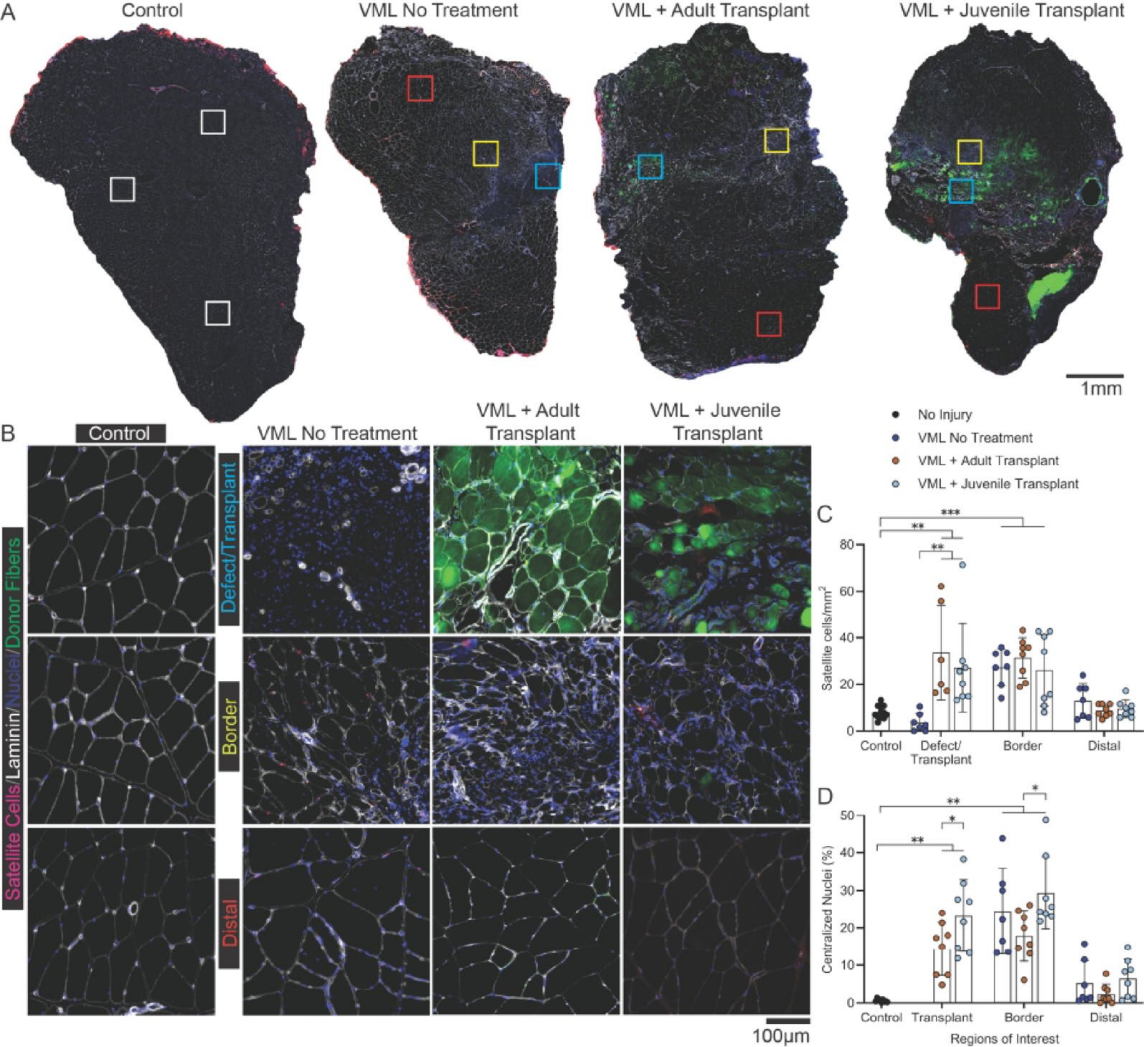
Satellite cell density and central nuclei across regions of VML-injured muscle following transplantation. (**A**) Representative whole-muscle cross-sections from uninjured controls, VML No Treatment, VML + Adult Transplant, and VML + Juvenile Transplant groups. Colored boxes indicate regions of interest sampled for satellite cell analysis and centralized nuclei, including uninjured tissue (white), the VML defect and transplantation zone (light blue), the border region surrounding the defect (yellow), and distal muscle remote from the injury (red). High-magnification images in (**B**) display Pax7⁺ satellite cells (Pink) localized to the periphery of laminin-stained myofibers (grey), with DAPI (dark blue) used for nuclear counterstaining. GFP (green) marks donor tissue in transplant groups. (**C**) Quantification of satellite cell density (Pax7⁺ cells/mm²) showed ~8 cells/mm² in control muscle. The defect/transplant region of No Treatment animals did not differ from controls, whereas both adult and juvenile transplant groups displayed significantly higher satellite cell density compared to both controls and the No Treatment defect region (*p* < 0.001). Border regions exhibited a robust ~3-fold increase relative to controls across all injured groups (*p* < 0.001) but did not differ from each other. Distal regions remained comparable to control values (*p* >0.05). No difference was observed between adult and juvenile transplant groups within any region. (**D**) Centralized nuclei (% of fibers) were negligible in control muscle (<1%) but elevated in all injured groups. Within the transplant region, adult transplants contained ~15–21% centralized nuclei, whereas juvenile transplants showed a significantly greater percentage (~20–23%; *p* = 0.038 vs. adult). Border regions contained ~15–30% centralized nuclei, with juvenile transplants exhibiting the highest values (~25–30%) compared to adult (~15–21%; *p* = 0.033 vs. adult). Distal regions remained low across all groups, including ~6% in No Treatment, ~4–6% in adult transplants, and ~6–7% in juvenile transplants (*p* >0.05 vs. control). Centralized nuclei were not quantified in the defect region of untreated muscles due to the general absence of myofibers. Data are presented as mean ± SD. Statistical significance: *p<0.05, **p<0.001. Scale bars = 1 mm (**A**), 100 μm (**B**)

To assess the regional distribution of satellite cells following VML injury and transplantation, Pax7⁺ cells were quantified across three defined regions of interest: defect/transplant, border, and distal muscle (Fig. 6A-C). In uninjured controls, satellite cells were evenly distributed (8.0 ± 1.0 cells/mm²). In No Treatment animals, the defect region showed a modest decline in satellite cell density (3.6 ± 2.3 cells/mm²) that was not significantly different from controls, while the border region exhibited a significant increase (27.2 ± 8.3 cells/mm², *p* < 0.001 vs. control) and distal muscle remained unchanged (12.9 ± 5.3 cells/mm², *p* > 0.05).

Following transplantation, both adult and juvenile groups showed significantly higher satellite cell density within the defect/transplant region compared to both control and No Treatment muscles (adult: 33.6 ± 14.9 cells/mm²; juvenile: 27.1 ± 13.2 cells/mm²; *p* < 0.01 vs. control and No Treatment). Border regions were similarly elevated in all VML groups (~ 25–35 cells/mm²; *p* < 0.001 vs. control) but did not differ between treatment conditions. Distal muscle regions remained comparable to control values (~ 7–13 cells/mm²; *p* > 0.05). No significant differences were observed between adult and juvenile transplants in any region.

#### Centralized nuclei

The prevalence of centralized nuclei, an indicator of regenerating myofibers, was negligible in uninjured control muscle (0.6 ± 0.1%) but elevated across all VML-injured groups (Fig. 6D). In No Treatment animals, centralized nuclei were most abundant in the border region (27.9 ± 4.6%) and lower in distal muscle (5.8 ± 1.2%). Following transplantation, similar regional patterns were observed, with centralized nuclei enriched within the transplant and border regions and minimal distally. In adult transplant recipients, centralized nuclei comprised 15.0 ± 2.8% of fibers in the transplant region, 20.7 ± 3.1% in the border, and 4.4 ± 1.0% distally. Juvenile transplant recipients exhibited higher percentages than adults, with 23.3 ± 4.2% in the transplant region and 29.3 ± 5.0% in the border (*p* < 0.05 vs. adult), while distal muscle remained low (6.5 ± 1.4%) and not different from controls (*p* > 0.05). Centralized nuclei were not quantified in the defect region for the No Treatment group due to the limited number of fibers in that region.

Of note, because cytosolic GFP is water-soluble, aqueous steps during the immunohistochemistry process can minimize GFP fluorescence, a process often referred to as “leaching”. To avoid over-interpretation, quantifications of GFP⁺ area, GFP⁺ fiber CSA, satellite cell and central nuclei counts related to GFP areas were performed only on sections with visually detectable GFP signal; samples where GFP was not clearly detected were excluded from GFP-specific analyses but were used for all other outcomes.

## Discussion

This study provides the first direct in-vivo comparison of juvenile versus adult skeletal muscle transplants for the treatment of VML. We found that juvenile donor tissue possessed markedly higher satellite cell density, smaller myofibers (allowing the transfer of more fibers per area), and enhanced in-vitro differentiation compared to adults. Despite these cellular advantages, both juvenile and adult transplants restored myofiber number and partially improved muscle strength to a similar extent seven weeks after a VML injury. Functional recovery remained incomplete in both groups, and donor fibers were predominantly small at this time point. These findings indicate that donor muscle age influences intrinsic regenerative properties but do not necessarily translate into superior short-term, in-vivo outcomes in the absence of additional regenerative cues. Importantly, the current study represents an early post-transplantation window, and it remains possible that donor-age effects could emerge over longer periods.

In Study 1, our developmental analysis confirmed well-established age-related trends in muscle biology: nuclear and satellite cell densities were highest in juvenile muscle and declined progressively with maturation, consistent with prior studies in rodents and humans [10–12]. The smaller fiber CSA and higher myogenic differentiation of juvenile myoblasts support the concept that this tissue retains a more growth-permissive phenotype, which has been linked to increased adaptability and regeneration in other contexts [21, 22]. These properties provided a strong rationale for testing juvenile muscle as a donor source in VML repair.

It is also worth noting that despite our juvenile, adolescent, and adult myoblasts being derived from donors of different ages, the myoblasts were allowed to expand in culture for 5–7 days prior to use (passage 1). This initial expansion period could have allowed the cells to adapt to the culture conditions and “reset” their phenotype. Thus, the observed similarities in proliferation and significant, yet mild improvement in differentiation across age groups may underestimate the functional advantages of juvenile myoblasts.

In Study 2, both juvenile and adult transplants integrated into the defect site, contributed GFP^+^ donor fibers, and restored fiber counts to that of controls. Functional gains relative to untreated VML ranged from 35 to 50%, comparable to improvements reported in previous autologous or minced muscle transplant studies [7, 8]. The lack of significant differences between juvenile and adult groups may be explained by several factors. First, the juvenile advantage in satellite cell number may have been transient; by seven weeks, many donor satellite cells could have differentiated into myofibers, leading to normalization between groups. Second, the immature size of donor fibers in both groups suggests that hypertrophy was still incomplete at this time point, limiting functional recovery. Finally, the restrictive microenvironment of the VML defect, previously characterized by altered extracellular matrix composition, persistent denervation, and limited vascularization [2, 5, 6], may have constrained the integration of juvenile tissue’s full regenerative potential.

Satellite cell analyses support this interpretation. In both donor age groups, Pax7⁺ cells were primarily located within the transplant and border regions, which were no different than the border region in the no treatment group, suggesting no apparent expansion into the surrounding host muscle. This pattern is consistent with prior evidence that satellite cells remain confined to areas containing intact basal lamina and supportive extracellular matrix [18, 23]. Without additional cues, migration into the broader defect is limited, which may have contributed to the absence of greater regenerative effects.

Centralized nuclei findings further support this view. Both transplant groups demonstrated localized enrichment of regenerating fibers within the transplant and border regions, whereas distal muscle remained largely unaffected. Juvenile recipients exhibited higher proportions of centralized nuclei in both the border and transplant regions compared to adult recipients, suggesting more robust regenerative activity. However, these differences did not correspond to superior muscle size or force at seven weeks, indicating that the regenerative process was still incomplete and may require longer time frames or additional cues to translate into functional benefit.

From a translational perspective, these results underscore that donor tissue composition, while important, is only one determinant of transplant efficacy. The persistence of small-diameter fibers and limited functional recovery in both transplant groups suggest that mechanical loading and/or pro-hypertrophic stimuli may be needed to maximize transplant benefit [18–20]. Rehabilitation strategies such as voluntary wheel running, resistance exercise, or electrical stimulation have been shown to enhance myofiber hypertrophy, satellite cell activation, and neuromuscular integration after injury [19, 20]. Incorporating such interventions into future transplant protocols could amplify the intrinsic advantages of juvenile-like donor tissue.

This work has several limitations. First, we assessed the regenerative outcomes using a single time point of seven weeks. While this was intended to capture the regenerative window for muscle injury, it could be too early to detect long-term integration of the transplant, such as hypertrophy, reinnervation, and extracellular matrix remodeling. A longer follow-up may reveal whether age-related advantages or transplant integration change as fibers mature. Second, we deliberately implemented a transplant-only strategy to isolate donor-age effects under controlled conditions. This baseline now creates a platform to test whether targeted loading (rehabilitation) or additional biological cues can enhance the benefits of juvenile tissue. Third, donor integration was quantified using GFP-based histology, which is susceptible to leaking; future work could incorporate other tracking methods, such as tissue clearing with 3D quantification. Finally, experiments were performed in male Lewis rats to mirror the male predominance of combat-related VML; confirming these findings in females and across additional genetic backgrounds will be an important step toward broad translation.

## Conclusion

Juvenile skeletal muscle exhibits cellular and structural features indicative of high regenerative potential, including abundant satellite cells and enhanced in-vitro differentiation. In this study, however, both juvenile and adult transplants integrated into volumetric muscle loss defects, restored myofiber number, and produced comparable functional recovery at seven weeks post-injury. These findings reinforce that muscle transplantation is a promising approach for restoring structure after VML, but optimizing donor age alone is insufficient for complete recovery within this early time frame. Longer-term studies are needed to determine whether donor age influences outcomes as transplanted fibers mature and remodel.

In addition to longer-term follow-up and the inclusion of rehabilitative exercise, future work will investigate how microenvironmental factors influence transplant integration, including extracellular matrix remodeling, innervation of donor fibers at neuromuscular junctions, revascularization of donor tissue, inflammatory signaling, mitochondrial function and the regulation of regenerative signaling pathways. These analyses will help clarify how donor tissue interacts with the host environment and may identify additional targets for enhancing long-term transplant efficacy.

## Data Availability

The datasets generated and/or analyzed during the current study are available from the corresponding author on reasonable request.

## Abbreviations

CSA: Cross-sectional area
DAPI: 4′,6-Diamidino-2-phenylindole
EDL: Extensor digitorum longus
FBS: Fetal bovine serum
GFP: Green fluorescent protein
IACUC: Institutional Animal Care and Use Committee
IHC: Immunohistochemistry
MyHC: Myosin heavy chain
NGS: Normal goat serum
Pax7: Paired box protein 7
PBS: Phosphate-buffered saline
TA: Tibialis anterior
VML: Volumetric muscle loss
WGA: Wheat germ agglutinin
